# Benefits of Outcomes of the Microscopic Examination of Anastomotic Donuts After Colorectal Resection for Oncological Purposes: A Medical Record-Based Study

**DOI:** 10.7759/cureus.7932

**Published:** 2020-05-02

**Authors:** Ihtisham Haq, Osama Shakeel, Awais Amjad, Faizan Ullah, Hannan Ali, Aun Jamal, Shahid Khattak, Aamir Ali Syed

**Affiliations:** 1 Surgical Oncology, Shaukat Khanum Memorial Cancer Hospital and Research Center, Lahore, PAK; 2 Surgical Oncology, Shaukat Khanum Memorial Cancer Hospital and Research Centre, Lahore, PAK; 3 Surgery, Shaukat Khanum Memorial Cancer Hospital and Research Center, Lahore, PAK; 4 Surgical Oncology, Shaukat Khanum Memorial Cancer Hospital and Research Center, Karachi, PAK; 5 Surgical Oncology, Shaukat Khanum Memoiral Cancer Hospital and Research Centre, Lahore, PAK

**Keywords:** colorectal cancer, donuts, coloanal anastamosis, anterior resection, margins

## Abstract

Objective

The objective of the study is to investigate the benefits of pathological assessment of donuts removed during coloanal anastomosis after anterior resection.

Methodology

During three years, 220 patients underwent circular stapled anastomosis. It is a retrospective study with convenient sampling. Involvement of donuts, the involvement of margins, length of donuts, and margins were primarily recorded. Ethical review approval was taken from the Institutional Review Board. Hospital electronic system was used to retrieve the data.

Results

Two hundred and twenty patients underwent circular end to end anastomosis (CEEA) stapled gun anastomosis. All had adenocarcinoma. Most of the patients had T3 disease (n=113). Low anterior resection was the most common procedure followed by anterior resection and sigmoid colectomy, respectively. We performed all rectal cancers anastomosis with a circular stapling gun. On histological analyses among 220 patients, only two patients were found to have a positive distal donut. No proximal donuts were positive. Both patients were also found to have positive distal margins. The mean length of the proximal donut was 1.79±0.45 cm. The mean length of the distal donut was 1.68±0.48 cm. Two distal margins and none of the proximal margins were positive for cancer. The mean length of the proximal margin was 8.69±4.48 cm. The mean length of the distal margin was 4.9±5.98 cm. Both patients had already received six months of pre-operative chemoradiotherapy and were not offered any additional treatment. Both patients were kept on close surveillance.

Conclusion

Routine analyses of the donuts after anterior resection has no impact on the management and outcome of the disease.

## Introduction

Colorectal cancer is the third most common cancer and the fourth leading cause of death in the world, accounting for 1.4 million new cases worldwide [1]. The incidence of colorectal cancer in Pakistan ranges from 4% to 6.8%, and it is on the rise [2]. Surgery remains the mainstay of treatment, and chemo-radiotherapy can be used as an adjunct therapy.

The difficulty of hand-sewn end to end anastomosis has been significantly reduced with the advent of circular stapling guns, especially in the deep pelvis [3, 4]. Circular end to end anastomosis (CEEA) stapling gun was first described in 1979 [5]. The next year, Griffin and Knight described a different way of rectal resections by closing the distal stump with a linear stapler and using a circular stapler for anastomosis (double stapling technique) [6]. First, the resection of the tumor is performed to get a negative margin; then linear stapler is used to close the distal stump. An anvil is introduced in the proximal segment of the bowel. CEEA stapling gun is introduced from the anal canal, and hence an anastomosis is performed. This device has been beneficial in performing anastomosis safely and efficiently, especially in the lower rectum. Minimally invasive surgeries, along with CEEA, have significantly increased the chance of sphincter sparing surgeries among patients with lower rectal cancers.

The use of CEEA for anastomosis resulted in reduced numbers of permanent colostomies [7]. Anastomosis with circular stapler yields proximal and distal donuts, which theoretically represent the true margins. It is a common practice to send these donuts for histopathological analyses [8]. Many guidelines recommend the microscopic analyses of proximal and distal margins as well [9].

The length of the distal margin after colorectal surgery always remains controversial. Newer studies advocate even margins less than 5 mm do not affect the outcomes adversely [10, 11]. This results in more sphincter sparing surgeries, however, at the cost of the increased risk of positive margins. In recent years, questions arose regarding the microscopic analyses of anastomotic donuts. Some studies suggest performing histological examination only if the distal margin is less than 2 cm, while other advocates there is no need for examination [12-14].

Recent studies suggested these examinations are time-consuming and expensive. Hence, our study aims to identify the efficacy of microscopic analyses of anastomotic donuts. The secondary endpoints are to identify when the microscopic analyses in warranted and the cost-effectiveness of the examinations.

## Materials and methods

From 1^st^ of January, 2015 to 31^st^ of December, 2018, all patients who underwent circular end to end anastomosis for colorectal cancer at Shaukat Khanum Memorial Cancer Hospital and Research Center, Pakistan were selected. The exemption was sought from the Institutional Review Board (IRB) as no intervention was involved, and our study was focused only on data extraction and analysis. The study was registered in a Chinese Clinical Trial Registry with number ChiCTR1900026482. Data was collected through a prospectively maintained database.

All reporting in our hospital is done according to the American College of Pathologists reporting guidelines for colorectal cancer. It is our standard practice to examine the donuts produced after stapled anastomosis, which are then examined by consultant pathologists. Patients who underwent abdominoperineal resection and permanent colostomy were excluded due to the unavailability of donuts. Variables recorded were age, gender, circumferential resection margin (CRM), clinical T stage, the involvement of proximal and distal margins, the involvement of proximal and distal donuts, length of proximal and distal margins, length of proximal and distal donuts. Margins are found to be involved if the tumor was found within 1 mm of margin. If there was any involvement of margins, the actions taken to tackle the condition were also recorded.

Calculations were performed with Statistical Package for the Social Sciences version 20.0 (IBM Inc., Armonk, US). Data were described using the median with minimum and maximum value for the skewed distribution of quantitative variables. For categorical variables, the number of observations and percentages were reported. 

## Results

A total of 220 patients were included in the study who underwent colorectal resections. One hundred and thirty patients were male (59%), and 90 patients were female (31%) with a ratio of 1.44:1. The median age at diagnosis was 51 years (ranged from 20 to 87 years). The histopathology of all resection specimens was adenocarcinoma. Out of 220 patients, low anterior resection (n=112), anterior resection (n=46), sigmoid colectomy (n=46), pelvic exenteration (n=6), left hemicolectomy (n=4), Hartman procedure (n=4) and total colectomy (n=2) were performed. As per the T stage, the majority of the patients were T3 (Table 1).

**Table 1:** T stages of tumor

T stage of cancer	Frequency (n=202)
T0	26 (11.8%)
T1	10 (4.5%)
T2	32 (14.5%)
T3	118 (53.6%)
T4	32 (14.5%)

On histological analyses among 220 patients, all the proximal donuts were negative, and among distal, only two donuts were positive. Both patients also had positive distal margins. The mean length of the proximal donut was 1.79±0.45. The mean length of the distal donut was 1.68±0.48. All of the proximal margins were negative for cancer involvement. The mean length of the proximal margin was 8.69±4.48. All the distal margins were also negative. The mean length of the distal margin was 4.9±5.98 cm (Table 2).

**Table 2:** Statistics of donuts

All patients	Mean (cm)	Median (cm)	Shortest distance (cm)
Proximal donut	1.79	1.80	0.80
Distal donut	1.67	1.50	0.50
Proximal margin	8.69	8	2.5
Distal margin	4.92	4	0.0

## Discussion

Our study advocates that routine examination of donuts does not benefit the outcomes. Two of our patients had positive distal donuts but also positive distal margins meaning thereby that the assessment of donuts didn’t offer any additional benefit. Many recent studies echoed the same findings [14,15]. Hence, unnecessary resources are utilized in examining the donuts. Not sending the donuts for histopathological examination resulted in a saving of approximately $5,000 per annum.

The appropriate length of the distal margin is still in debate among professionals. The longitudinal intraluminal spread of cancer greater than 1 cm from the macroscopic tumor site is a rare phenomenon [16]. Hughes and Jenevein found the intramural spread of 2 cm in only 4.7% of the cases [17]. Sidoni found the intramural spread of less than 2 cm in all cases except one case [18]. Shimada demonstrated the intramural spread of at least 1 cm in rectal cancer patients [19]. Therefore, in the recent few years, the appropriate length of distal margin has reduced, and a margin of 2 cm is considered adequate to achieve a negative margin. We make sure that we take a minimum of 2 cm gross margin for low rectal cancers and an even large margin for higher tumors. In our study, 39 patients had a distal resection margin of less than 2 cm, with two of them having a positive margin. However, this didn’t result in any change in their management plans since all our patients already complete a long course of neoadjuvant chemoradiotherapy and are not offered any adjuvant therapy.

The incidence of carcinoma found in donuts after resection is 0.5-0.8% [12-15]. In our study, only two of our specimens were marked positive for carcinoma after microscopic investigation, and these patients also had positive margins, which means thereby that the assessment of donuts didn’t have an impact on changing management plans. However, there are certain situations where a microscopic analysis is warranted. Cancers with aggressive behavior such as small cell carcinoma, pure signet ring cell carcinoma, undifferentiated cancers, cancers with the infiltrative patterns, or with extensive lymphovascular invasion should undergo the microscopic examination of anastomotic donuts [16, 20-23]. Some studies have also recommended performing analysis of donuts only if the distal margin is less than 2 cm on microscopic examination. Our study, however, didn’t show any benefit even for patients with positive donuts as their margins were also positive.

Our hospital is a charity based cancer organization. As such, we should establish local guidelines as to which patients merit an analysis of donuts. This will result in a significant saving of cost and time. One strategy would be to send the donuts along with primary surgical specimens and only have the donuts examined if the margin is positive. This strategy has also been endorsed by the American College of Pathologists [24]. Although studies have neglected the fact that positive distal margin with a negative distal donut doesn’t offer any advantage because a donut isn’t a true representative of the true margin. The fact that the donut is negative could only mean that the donut is from some other part of the distal rectum and not a true representative of the margin.

## Conclusions

Routine examination of anastomotic donuts does not affect the outcomes and management of the disease. We recommend that donuts should be sent with the primary specimen and only be examined if the specimen margins are found positive. This will result in significant reduction in cost and time.
